# Levobuipivacaine-Induced Dissemination of A549 Lung Cancer Cells

**DOI:** 10.1038/s41598-017-08885-z

**Published:** 2017-08-17

**Authors:** Shun-Ming Chan, Bo-Feng Lin, Chih-Shung Wong, Wen-Ting Chuang, Yu-Ting Chou, Zhi-Fu Wu

**Affiliations:** 1 Department of Anaesthesiology, Tri-Service General Hospital, National Defence Medical Centre, Taipei, 114 Taiwan; 2 Graduate Institute of Medical Sciences, National Defence Medical Centre, Taipei, 114 Taiwan; 3 Anaesthetic and Analgesic Common Laboratory, National Defence Medical Centre, Taipei, 114 Taiwan; 4 Department of Aneasthesiology, Cathay General Hospital, Taipei, 106 Taiwan; 5 Insitute of Biotechnology, National Tsing Hua University, Hsinchu City, 300 Taiwan

## Abstract

While anaesthetics are frequently used on cancer patients during surgical procedures, their consequence on cancer progression remains to be elucidated. In this study, we sought to investigate the influence of local anesthetics on lung cancer cell dissemination *in vitro* and *in vivo*. A549 human non-small lung cancer cells were treated with various local anaesthetics including ropivacaine, lidocaine, levobupivacaine and bupivacaine. Cell barrier property was assessed using an electric cell-substrate impedance sensing (ECIS) system. The epithelial-to-mesenchymal transition (EMT) of treated cells was studied by immunofluorescence staining. *In vitro and in vivo* cancer cell dissemination were investigated.Gene expression microarray and quantitative real-time PCR (qrt-PCR) assays were used to identify the genes responsible for levobupivacaine-mediated cancer cell dissemination.The results illustrated that only levobupivacaine induced EMT in the treated cells and also caused the dissemination of cancer cells *in vitro*. In addition, after intravenous injection, levobupivacaine encouraged cancer cell dissemination *in vivo*. Gene expression microarray, qrt-PCR and immunoblotting revealed that after levobupivacaine treatment, the hypoxia-inducible factor (HIF)- 2α gene was upregulated in cancer cells. Our findings suggest that levobupivacaine may induce A549 lung cancer cell dissemination both *in vitro* and *in vivo*. More specifically, HIF-2α signaling possibly contributes to levobupivacaine-mediated A549 lung cancer cell dissemination.

## Introduction

Lung cancer is one of the most common causes of death worldwide and surgical resection remains the major treatment option of early-stage tumor^1^. However, metastasis and tumor recurrence after surgery represent major clinical challenges that are responsible for the low survival rate of lung cancer patients. Multiple risk factors in the perioperative period contribute to tumor cell proliferation and eventual metastasis^2^. Several studies have shown that frequently used local anaesthetics inhibit the invasion of cancer cells by blocking the sodium channel^3, 4^ while other studies have attributed the anti-metastatic ability of local anaesthetics to other relevant pathways^5, 6^. Furthermore, retrospective analysis of patients who underwent surgery for breast or prostate cancer suggests that regional anaesthesia reduces cancer recurrence and improve survival rate of patients^7–9^. In contrast, other observational studies of patients who underwent non-small lung cancer surgery have shown that epidural bupivacaine anaesthesia does not improve the overall survival rate^10, 11^. Thus, a clear relationship between local anaesthesia and the clinical outcome of patients with cancer is currently unestablished^12^.

Tumor invasion and metastasis can be attributed to epithelial to mesenchymal transition (EMT), which is the process in which epithelial cells lose epithelial characteristics and acquire migratory potential to become invasive^13^. Recent studies have suggested that moderate hypoxic conditions can trigger the EMT process, which in turn causes different human cancer cells to invade and become resistant to therapy^14, 15^. Hypoxia of the non-dependent lung where the cancer mass is located is often encountered during thoracic surgeries. This is further complicated by the fact that cancer patients are often immunosuppressive and surgical manipulation may contribute to tumor metastasis^16^. Hypoxia-inducible factors (HIF) are a family of transcription factors related to tumor progression and regulates both overlapping and unique downstream target genes involved in critical aspects of cancer activity such as cell proliferation, angiogenesis, glucose metabolism and cell invasion^17–20^. Currently, the expression of HIF-2α under hypoxic conditions are unclear. To the best of our knowledge, the triggering of EMT in human cancer cells by HIF-2α or other pathways after local anaesthetics has not yet been explored.

In the present study, we hypothesized that local anaesthetics induces EMT in lung cancer cells by activating HIF-2α. Due to the lack of relevant studies, the aim of this investigation was to discuss the influence of local anaesthetics on HIF-2α expression in lung cancer cells.

## Results

### Local anaesthetics reduced barrier function and induced EMT in A549 lung cancer cells

**Figure 1:**
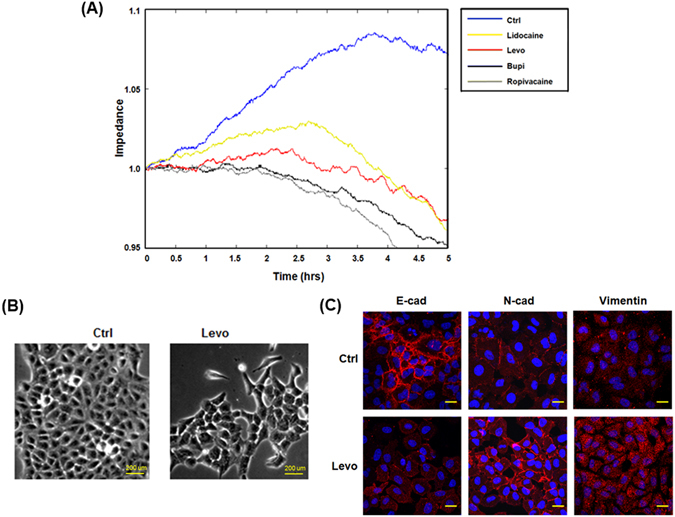
Effects of ropivacaine, lidocaine, bupivacaine and levobupivacaine on cell barrier function and EMT of A549 lung cancer cells. (**A**) Barrier function of lung cancer cells with and without local anaesthetics treatment as assessed by electric cell-substrate impedance sensing (ECIS). Measurements were performed at 4 kHz. (**B**) Effect of levobupivacaine on the morphology of A549 cells. Levobupivacaine treatment induced morphological change after 24 hr exposure. (**C**) Immunofluorescence staining of E-cadherin (E-cad), N-cadherin (N-cad) and vimentin for A549 cells treated with or without levobupivacaine. Ropi indicates ropivacaine; Lido indicates lidocaine; Bupi indicates bupivacaine, Levo indicates levobupivacaine; and Ctrl indicates control. Scale bar = 200 μm.

The first step of cancer cell dissemination involves the attenuation of barrier function. To examine the effect of anesthetics on the barrier function of lung cancer cells, A549 and H1975 cells were treated with various local anaesthetics and subjected to ECIS system analysis (Fig. 1A and Supplementary Figure S1). Levobupivacaine, lidocaine, ropivacaine and bupivacaine all reduced the barrier property in A549 lung cancer cells, but not H1975 cells. Furthermore, while levobupivacaine treatment induced morphological change in A549 cells from cuboidal to spindle-like (Fig. 1B), the same was not observed after lidocaine, ropivacaine and bupivacaine treatments. On the other hand, as determined by immunofluorescence staining, levobupivacaine treatment reduced the level of E-cadherin, an epithelial marker, and increased the expression of N-cadherin and vimentin, a mesenchymal marker (Fig. 1C). The enhancement of vimentin and N-cadherin expressions indicate EMT in A549 cells following levobupivacaine treatment^21^. In contrast, EMT was not observed in cells after lidocaine, ropivacaine and bupivacaine treatments.

### Levobupivacaine induced A549 lung cancer cell dissemination i*n vitro* and *in vivo*

**Figure 2:**
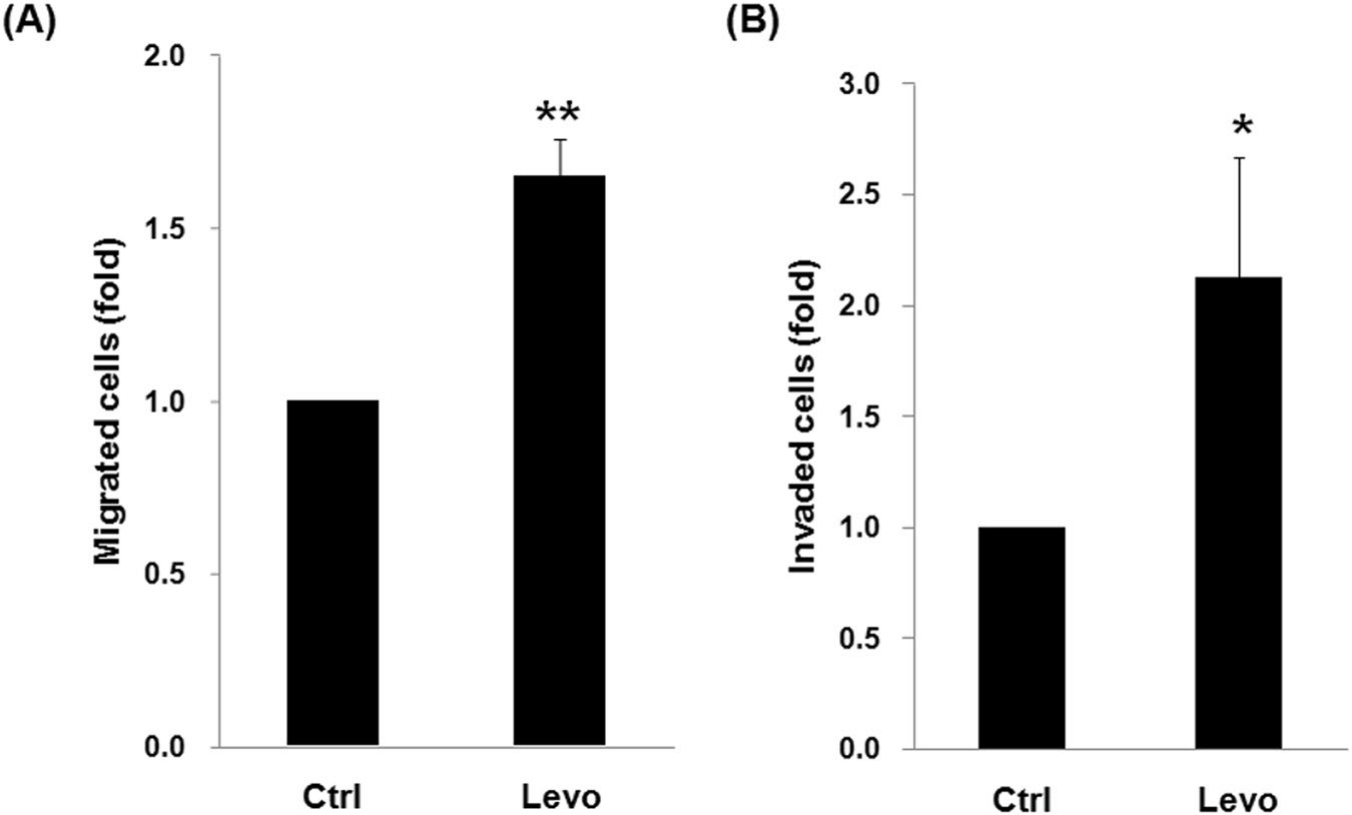
Levobupivacaine induced migration and invasion in lung cancer cells. (**A**) Transwell migration test for A549 cells treated with or without levobupivacaine. Migratory cells were quantified. Each column represents the mean ± SD **P < 0.01. (**B**) Transwell invasion test for A549 cells treated with or without levobupivacaine. Invading cells were quantified. Each column represents the mean ± SD *P < 0.05. Levo indicates levobupivacaine and Ctrl indicates control.

**Figure 3:**
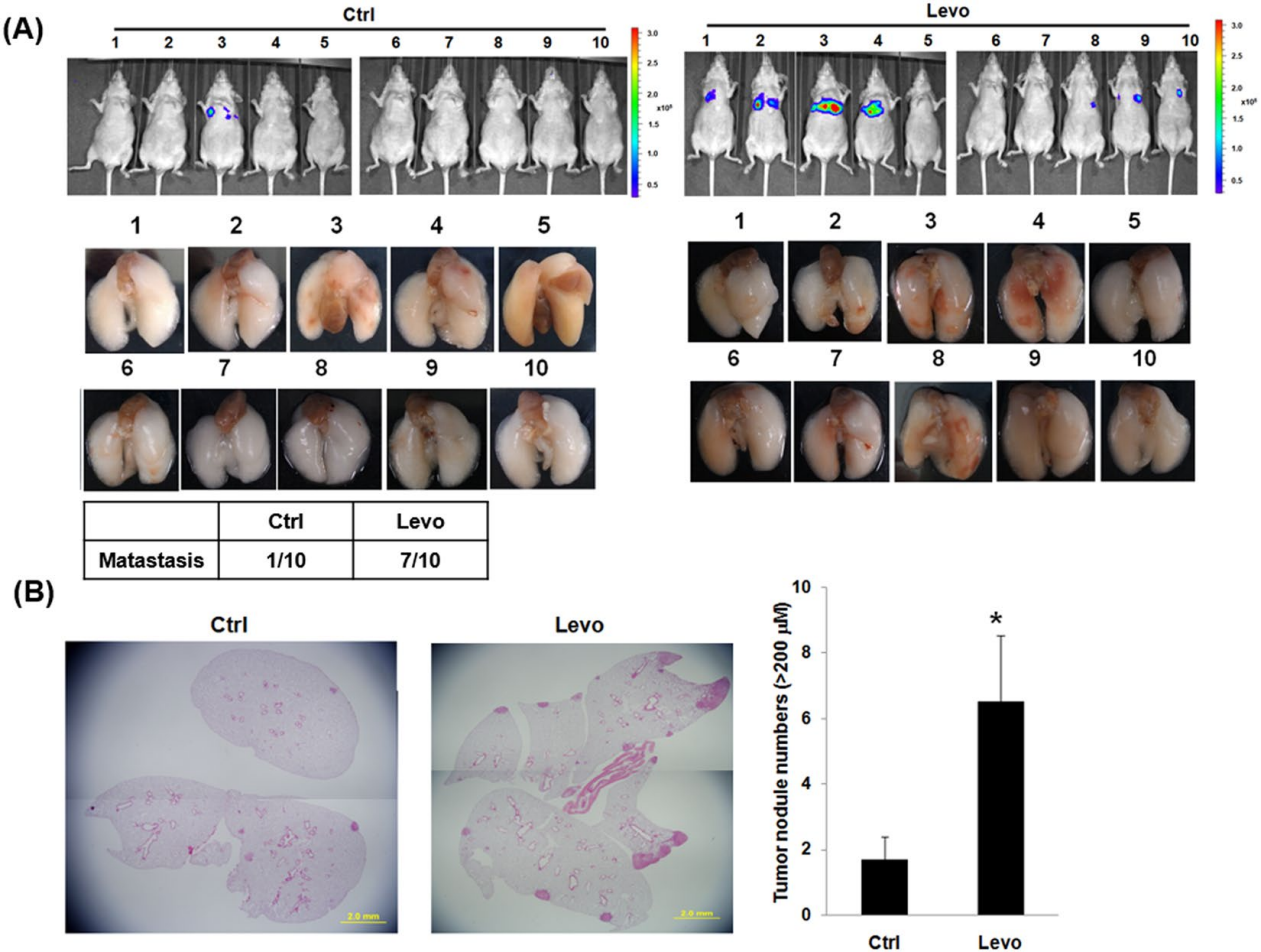
Levobupivacaine encouraged cancer cell dissemination *in vivo*. (**A**) IVIS imaging performed on nude mice after i.v. injection of A549luc cells treated with or without levobupivacaine (upper). Photographs of lungs harvested from xenografted mice (lower) (Day 86). (**B**) H&E staining for micro- or macro-nodules in the lungs of xenografted mice (left). Quantitative analysis for tumor nodules in the lungs of xenografted mice (right). Histologic examination revealed a higher proportion of tumor nodules in the levobupivacaine group than in the control group. Furthermore, the experimental group was associated with bigger tumor size. Levo indicates levobupivacaine and Ctrl indicates control.

As EMT contributes to cancer cell migration and invasion, the effects of levobupivacaine on cell migration and invasion *in vitro* were studied. Levobupivacaine treatment was found to encourage both cancer cell migration (Fig. 2A) and cancer cell invasion (Fig. 2B). For the *in vivo* study, cancer cell dissemination into the lungs of immunodeficient mice was monitored using IVIS after the injection of A549luc cells pre-treated with levobupivacaine. Promotion of cancer cell dissemination into the lungs of xenografted mice was observed, as depicted in Fig. 3A. Staining of harvested lungs from xenografted mice revealed significantly more tumor nodules after the injection of levobupivacaine-treated cells than control cells (Fig. 3B). These results altogether suggest that levobupivacaine treatment encourages A549 cancer cell dissemination *in vivo*.

### Levobupivacaine upregulated HIF-2αexpression

**Figure 4:**
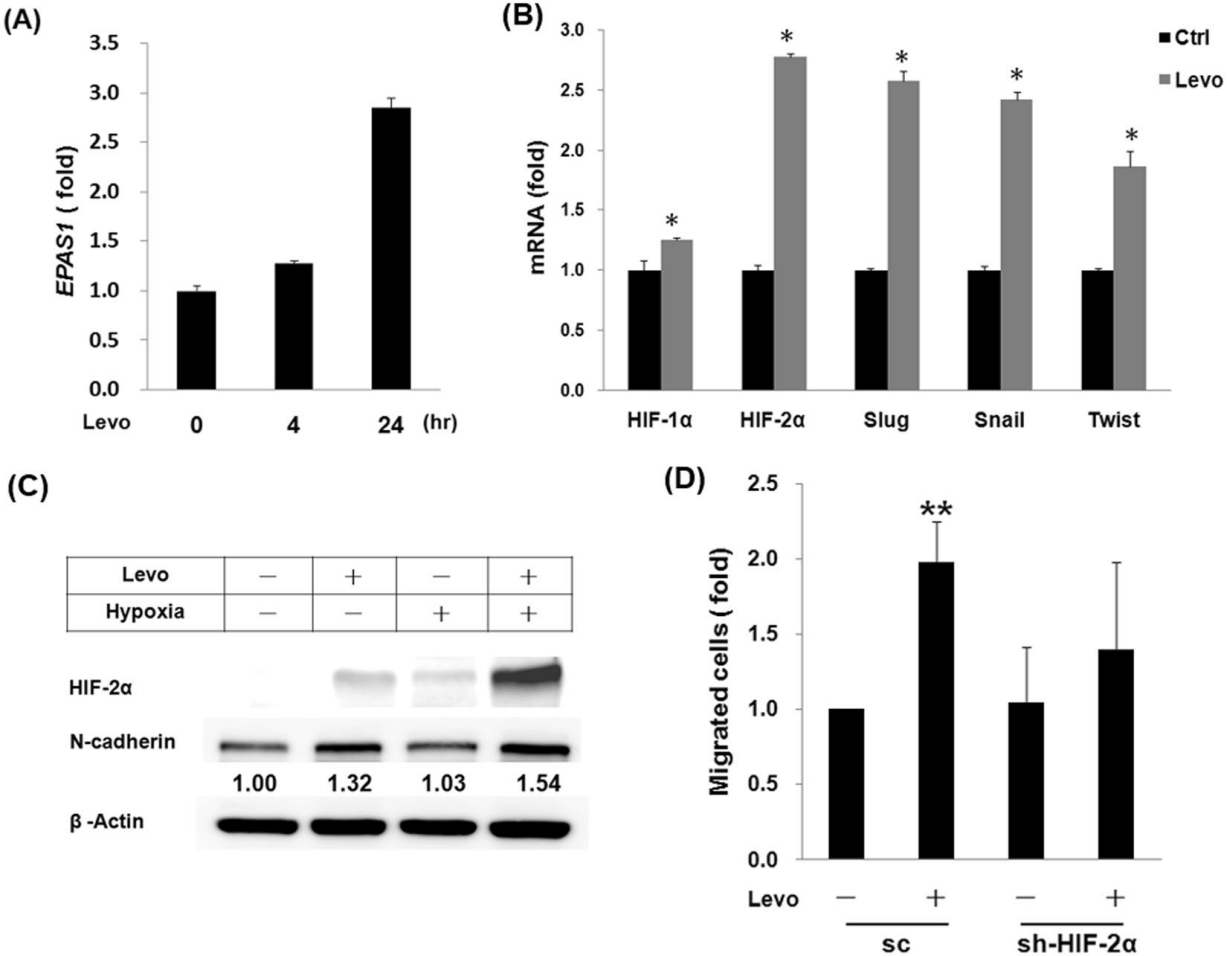
Levobupivacaine induced HIF-2α expression. (**A**) Q-PCR analysis of HIF-2α transcripts extracted from A549 cells treated with levobupivacaine for indicated periods. (4 and 24 hrs) (**B**) Q-PCR analysis to determine mRNA levels of HIF-1α, HIF-2α, Slug, Snail and Twist in A549 cells treated with or without levobupivacaine for 24 hrs. (**C**) Immunoblotting analysis for HIF2-α and N-cadherin expression in A549 cells under hypoxia and/or levobupivacaine treatment. (**D**) Transwell migration analysis for A549 cells infected with lentiviral RNA against HIF-2α (shHIF-2α) or Scramble (SC) followed by treatment with or without levobupivacaine. Levo indicates levobupivacaine and HIF indicates hypoxia-induced factor. EPAS1 (Endothelial PAS domain-containing protein 1 is also known as hypoxia-inducible factor-2alpha (HIF-2α).

Gene expression microarray analysis and Q-PCR identified HIF-2α as a levobupivacaine-inducible gene and levobupivacaine induced EMT-regulatory genes (Fig. 4A and B). Immunoblotting further revealed that levobupivacaine potentiated hypoxia-induced expressions of HIF-2α and N-cadherin (Fig. 4C). Furthermore, knockdown of HIF-2α attenuated levobupivacaine-induced cancer cell migration (Fig. 4D). In summary, the data suggest that HIF-2α may contribute to levobupivacaine-induced cancer dissemination.

## Discussion

This study began with an elucidation of the influence of levobupivacaine, lidocaine, ropivacaine and bupivacaine on A549 and H1975 cell barrier property and found that while levobupivacaine significantly increased A549 migration and invasion, but not in H1975 cells. The same was observed after ropivacaine, lidocaine, and bupivacaine treatments in A549 cells. Furthermore, in addition to *in vitro* EMT, migratory consequence was observed *in vivo* after levobupivacaine treatment. On the genetic level, HIF-2α overexpression was noted after levobupivacaine exposure. Regarding these observations, we propose a causal relationship between levobupivcacaine and EMT which is mediated by HIF-2α induction.

Dynamic monitoring via ECIS technology was used for rapid assessment of cell invasive capacity. By applying this new approach, important features of cellular response such as attachment, spreading, migration, proliferation and differentiation can be observed in real-time with high sensitivity^22^. Thus, this method has been popularized for studying cellular behaviors in response to different drugs^23^. To the best of our knowledge, this study is a pioneer research on local anaesthetics-related tumor invasion in local anaethetics field. Reduced impedance of monolayers treated with anaesthetics can be attributed to invasion, extravasation, or motility. Lidocaine, bupivacaine, ropivacaine and levobupivacaine-treated cells experienced more significant impedance reduction than control cells which suggests increased invasiveness due to greater A549 viability. However, after levobupivacaine treatment, notable morphological change in cells from cuboidal to spindle-liked was observed. The migration and invasion potentials of levobupivacaine-treated A549 were further confirmed via migration and invasion transwell tests. Taken together, these results emphasize the role of levobupivacaine in inducing invasion capacity of the A549 cell line.

Despite continuous concerns that surgery may compromise the patient’s immune defense and thus facilitate tumor metastasis, surgical tumor removal remains the most suggested treatment for lung cancer patients. In a review article, Heaney *et al*. explained that lower cancer recurrence rates associated with local or regional anaesthesia can be attributed to the better preserving of patient immunity in comparison to general anesthesia^24^. Additionally, postoperative epidural anaesthesia with local anaesthetics has been shown to reduce perioperative and postoperative stress and pain intensity. Other direct outcomes of local anaesthetics on tumor cells include inhibited tumor invasion and suppression of metastatic ability^25, 26^. A previous study has demonstrated the significant upregulation of the sodium channel during breast cancer progression which potentiates a series of cell behaviors integral to metastasis^3^. Chang *et al*. also showed that lidocaine and bupivacaine induced apoptosis in breast tumor cells and exerted antitumor effect^27^. However, the findings of this study are in disagreement with previous studies that associated local anaesthetics with antitumor effect^5, 6^. This could in part be due to the use of bupivacaine and lidocaine in previous studies instead of levobupivacaine. In this study, levobupivacaine was found to significantly increase cell migration and invasion in A549 cells.

Besides, this study was conducted *in vivo* to investigate the effect of levobupivacaine on cancer cell dissemination after their introduction. IVIS analysis showed that levobupivacaine treatment promoted cancer cell dissemination into the lungs of xenografted mice (7/10 in the levobupivacaine treatment group compared to 1/10 in the control group). Histological sections further revealed more significant nodule metastasis in the levobupivacaine treatment group than the control group. One-lung ventilation is required in most lung cancer surgeries and is typically associated with hypoxemia and even hypoxia. Nevertheless, further studies are necessary in order to conclude the actual clinical effects of levobupivacaine in surgical conditions as well as the relationship among the drug, EMT, and clinical manifestation.

The role of HIF-2α in cancer metastasis has prompted the development of drugs that target the HIF pathway for lung cancer treatment^28, 29^. In addition, studies have linked the overexpression of HIF-2α to increased tumor size, invasion, progression and angiogenesis in non-small cell lung carcinoma^30–32^. Moreover, another study showed that the silencing of HIF-2α inhibited tumor growth in an A549 tumor model^33^. Recently, Bertout *et al*. suggested that HIF-2α likely contributes to tumor cell survival during radiation therapy^34^. In the present study, HIF-2α and EMT markers increased after levobupivacaine treatment. To coincide, the knockdown of HIF-2α in A549 decreased cell invasion and migratory ability. These results imply that levobupivacaine may contribute to EMT and modulate cell mobility by regulating HIF-2α. Under hypoxia, significantly higher HIF-2α expression was observed after levobupivacaine treatment than under normal condition. As previously suggested by Shimoda *et al*., the complex regulation of tumor growth and invasion are not simply controlled by the presence or absence of HIF-2α^35^. Lirk *et al*. presented that local anaesthetics associated with DNA demethylation in breast cancer cells^36^. Benzonana *et al*. presented that volatile anaesthetics to an HIF-α mediated mechanism of action facilitating renal cell cancer metastasis *in vitro* ^37^. These studies imply that the relation between anaesthetics and tumor metastasisis more complex than our recognition at present. Thus, the results of this study are a reminder that the understanding of the mechanisms underlying the effect of levobupivacaine on HIF-2α function is far from complete and requires additional evaluation.

There are limitations to this study. First, despite the clinical appropriateness of the drug dose used, *in vitro* studies are limited to model tumor response. For example, while a single dose of levobupivacaine was shown to promote metastasis of lung cancer cells *in vitro*, dosing parameters may differ *in vivo*. Ultimately, only a well-planned and executed *in vivo* research can accurately model the tumor environment and the delivery of anaesthetics. Second, the immunosuppression level, surgical techniques and anaesthetic regimens are all relatively complex and thus require more comprehensive consideration clinically. Third, because only two cancer cell lines were studied and cell lines do not completely mimic primary cells, more investigations are necessary to establish specifically the effects of different anesthetics on different cancer cell lines. Fourth, while this study directly exposed A549 to levobupivacaine, in clinical condition, levobupivacaine is typically introduced by the epidural route. Finally, in our mice model that A549 luc cells were levo-treated before injection.

In conclusion, our findings suggest that levobupivacaine may be responsible for the significant increase in EMT, which is mediated by the up-regulation of HIF-2α in the A549 lung cancer cell line. Our data suggests that HIF-2α may play a crucial role in the regulation of EMT, tumor migration, invasion and metastasis. Further studies are required to provide more solid evidence that can establish a relationship between levobupivacaine and the clinical outcome in lung cancer surgery in order to accentuate the importance of local anaesthetics in tumor recurrence and dissemination.

## Methods

### Reagents

Bupivacaine, lidocaine and ropivacaine were obtained from Sigma-Aldrich (St. Louis, MO, USA). Levobupivacaine was purchased from Abbott Laboratories Services Corp, Taiwan Branch (Taiwan). All other reagents used were of reagent grade.

### Cell line and culturing condition

For cell culture studies, human lung cancer cell line (A549&H1975) purchased from the American Type Culture Collection (ATCC, Manassas, VA, USA) was used. The cells were grown in RPMI-1640 medium supplemented with 10% fetal bovine as recommended by ATCC. The cells were grown in the monolayer at 37 °C in a humidified atmosphere with 5% CO_2_.

### Hypoxic treatment

The cells were placed in a polypropylene chamber connected to a supply of O_2_ and N_2_ gases. Oxygen gas concentration inside the chamber was continuously monitored using a sensor. The hypoxic cells were maintained in a controlled hypoxia chamber containing 1% O_2_, 5% CO_2_, and 95% N_2_ at 37 °C for 24 hrs.

### Impedance measurement via Electric Cell-Substrate Impedance Sensing (ECIS)

Electric cell-substrate impedance sensing (ECIS) 8-well electrode arrays were coated with 0.2% gelatin for 1 hr at room temperature. In order to stabilize the electrode array, a current source was supplied via a 1 V, 4000 Hz signal by the ECIS system (Applied BioPhysics, Inc) to 200 μL/well of culture medium. Cells were seeded at a density of 1.5 × 10^5^ cells/well and grown in 5% CO_2_ humidified environment at 37 °C after an equilibrium time of 2 hrs. Levobupivacaine, lidocaine, bupivacaine and ropivacaine were added at 1 mM, 8 μM, 1 mM and 1 mM respectively to 8-well electrode arrays (Applied BioPhysics, Inc) referred to previous studies^38–40^. The electrical impedance across the monolayer was measured after 24 hrs with ECIS at 1 V, 4,000 Hz. Resistance across monolayer was processed by ECIS software to obtain the impedance value.

### *In vitro* migration and invasion tests

Cell migration was studied using 24-well cell culture inserts (BD Biosciences). Transwell membranes 8 µm in pore size were pre-coated with gelatin at 100 μg/mL. Cells were then suspended in medium containing 10% Nu-serum and seeded into transwell chambers with or without levobupivacaine at a density of 5 × 10^4^ cells/well. After 5 hrs of incubation at 37 °C, invasive cells that migrated through the FluoroBlok membrane were stained with propidium iodine, visualized using a fluorescent microscope and counted with Image J software. For the cell invasion study, transwell membranes were pre-coated with Matrigel (5 mg/mL) and 2.5 × 10^4^ cells were added per well. After 24 hrs of incubation at 37 °C, cells were stained and counted.

### Immunocytochemistry

A549 cells were seeded in 6-well plates at a density of 1 × 10^5^ cells/well in complete growth medium and treated with either a 1 mM levobupivacaine solution or the vehicle control for 24 hrs. The expression levels of E-cadherin, vimentin and N-cadherin were visualized using an Olympus photo system (Olympus DP20 microscope camera, Japan).

### *In vivo* bioluminescence imaging

For animal studies, 20 male BALB/c nude mice were obtained from the National Laboratory Animal Center (Taipei, Taiwan) and kept in a conventional animal facility. All animal experiments were approved and conducted in accordance with the guidelines of the National Tsing Hua University Institutional Animal Care and Use Committee. Upon arrival, the animals were divided into two groups, the A549luc + Levo group and the A549luc control group. The luciferase expressing A549 cell line (A549luc) was purchased from Caliper Lifesciences Corp. After conventional expansion, A549luc cells (1.5 × 10^6^) were intravenously injected through the tail vein. A luciferin solution (2 mL, 150 mg/mL) (Firefly Luciferin, Caliper Lifesciences Corp, USA) was then injected intraperitoneally. Dissemination of fluorescent cells was probed using an *in vivo* imaging system (IVIS) (Caliper Lifesciences Corp, USA) every seven days for a period of three months. At each time point, animals were anesthetized using 2% isoflurane and imaged using a cooled CCD camera. Bioluminescent signals (photons/s) of xenografted mice were quantified using the Living Image 3.0 software (Caliper Lifesciences Corp, USA). After three months of monitoring, the animals were euthanized and pathological examinations were carried out.

### Histology

After euthanization, the lungs of the animals were removed, fixed immediately with 4% paraformaldehyde overnight at 4 °C and embedded in paraffin using an automatic tissue processor. For tissue slides, 20 μm thick tissue sections were cut using a microtome and stained with hematoxylin-eosin. The sections were inspected using an Olympus photo system. Lung metastases were counted as previously described and only those larger than 200 μm were considered.

### Quantitative real time-PCR

Total RNA was extracted using TRIZOL (Invitrogen) in accordance with the manufacturer’s protocol. Extracted RNA (2 μg) was reverse transcribed with random hexamer primers (Boehringer-Mannheim) using the Superscript II kit (Invitrogen). Relative gene expression was quantified using the 2^△△^C_T_ method on a LightCycler 480 real-time PCR System (Roche Applied Science, Indianapolis, IN, USA) together with the Universal Probe Library (Roche Applied Science).

### Microarray analysi**s**

After RNA preparation, microarray analysis was conducted by Genetech Biotech Bo., Ltd. (Taipei, Taiwan).

### Immunoblotting (Western blot analysis)

A549 cells were seeded in 10 cm dishes at a density of 1 × 10^6^ cells/plate and cultured with complete RPMI (Roswell Park Memorial Institute) medium for 24 hrs. Next, the cells were exposed to normoxic (20% O_2_) or hypoxic (1% O_2_) conditions for 24 hrs. Cells were then washed with PBS and treated with a modified RIPA lysis buffer that contained 20 mM Tris–HC1 pH 7.5, 150 mM NaCl, 1 mM EDTA, 1 mM EGTA, 1% (v/v) Triton X-100, 0.5% (v/v) Nonidet P40, 2.5 mM sodium pyrophosphate, 1 mM sodium orthovanadate, 50 mM sodium fluoride and 1x protease inhibitor cocktail. The crude lysate was left at −80 °C for 30 min and then transferred to a 4 °C environment to complete cell lysis. The sample was then centrifuged at 12,000 rpm for 20 min at 4 °C. The supernatant was transferred to a fresh tube and an aliquat was taken to determine the protein concentration using a DC (Detergent Compatible) protein assay kit (Bio-Rad). Proteins were separated using a traditional 10% gel via SDS-PAGE and then transferred to a nitrocellulose membrane (Millipore, USA). The membrane was subsequently blocked with blocking buffer (5% non-fat milk in TBS/Tween-20, TTBS) for 30 mins and incubated with antibodies overnight at 4 °C. Primary antibodies against HIF-2α (polyclonal, Abcam, USA) and N-cadherin (polyclonal, GeneTex, USA) were diluted 1000x prior to use. After treatment, the membrane was washed with TTBS and incubated with 5000x diluted horseradish peroxidase (HRP) conjugated goat anti-rabbit secondary antibody (GeneTex, USA) for 1 hr at room temperature. An enhanced chemiluminecsence (ECL) reagent (Millipore, USA) was used to develop the reactive bands which were quantified by densitometry using a Chemi Genius2 Bio Imaging System (Syngene, Cambridge, UK).

### Data analysis and statistics

All experiments were conducted in triplicate and statistical analysis was performed using the Student’s t-test, where observed values for each sample were studied in respect to the values obtained under normoxic/untreated conditions. A *p*-value of *p* < 0.05 was considered statistically significant.

## Electronic supplementary material

Supplementary Information 
