# Supplementary material for: Levobuipivacaine-Induced Dissemination of A549 Lung Cancer Cells
Source: Sci Rep. 2017 Aug 17;7:8646. doi: 10.1038/s41598-017-08885-z (PMC5561232; doi:10.1038/s41598-017-08885-z)

Levobuipivacaine-Induced Dissemination of A549 Lung Cancer Cells

Shun-Ming Chan[1,2,3], Bo-Feng Lin [1,2,3], Chih-Shung Wong [2,4], Wen-Ting Chuang[1,3], Yu-Ting Chou[5,*,+], Zhi-Fu Wu[1,2,3,*,+]

[1]Department of Anaesthesiology, Tri-Service General Hospital, National Defence Medical Centre, Taipei 114, Taiwan

[2]Graduate Institute of Medical Sciences, National Defence Medical Centre, Taipei 114, Taiwan

[3] Anaesthetic and Analgesic Common Laboratory, National Defence Medical Centre, Taipei 114, Taiwan

[4] Department of Aneasthesiology, Cathay General Hospital, Taipei 106, Taiwan

[5] Insitute of Biotechnology, National Tsing Hua University, Hsinchu City 300, Taiwan

[*] Co-corresponding authors

[+] these authors contributed equally to this work

Institution: Department of Anaesthesiology, Tri-Service General Hospital, National Defence Medical Centre, Taipei 114, Taiwan

Corresponding Author:

Zhi-Fu Wu

Department of Anaesthesiology, Tri-Service General Hospital, National Defence Medical Centre, Taipei 114, Taiwan

#325, Section 2, Chenggong Road, Neihu District, Taipei 114, Taiwan

Phone: +886-2-87927128

FAX: +886-2-87927127

Email: aneswu@gmail.com

SUPPLEMENTARY INFORMATION

Supplementary Fig.S1. Effects of ropivacaine, lidocaine, bupivacaine and levobupivacaine on cell barrier function of H1975 lung cancer cells

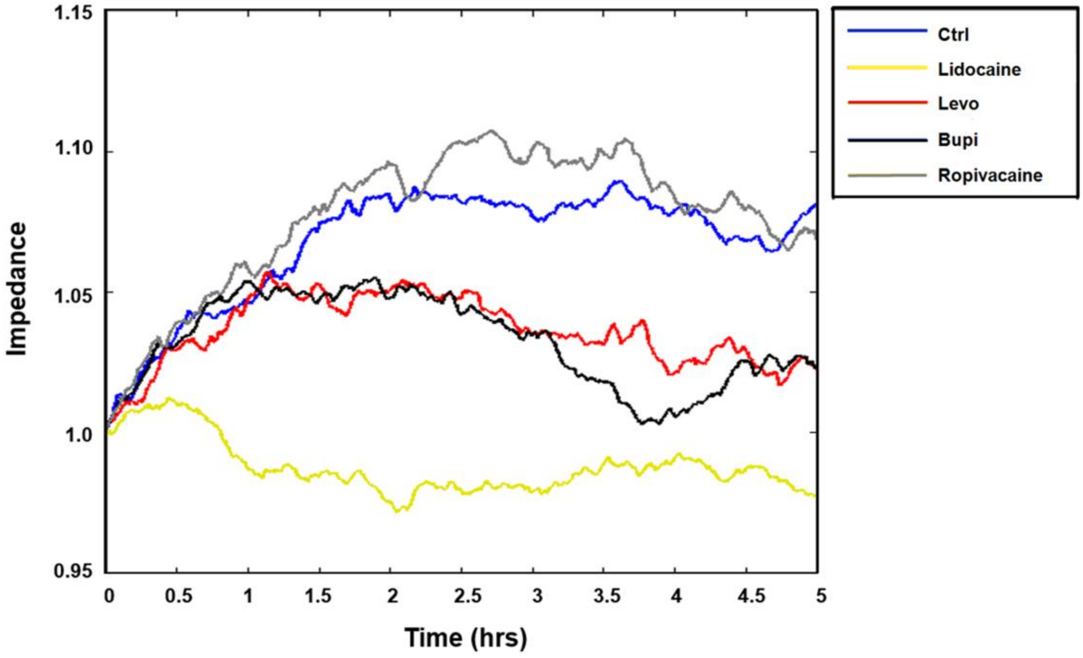

Barrier function of lung cancer cells with and without local anaesthetics treatment as assessed by electric cell-substrate impedance sensing (ECIS). Measurements were performed at 4 kHz.

Figure 4. (C) Full-length gels and blots

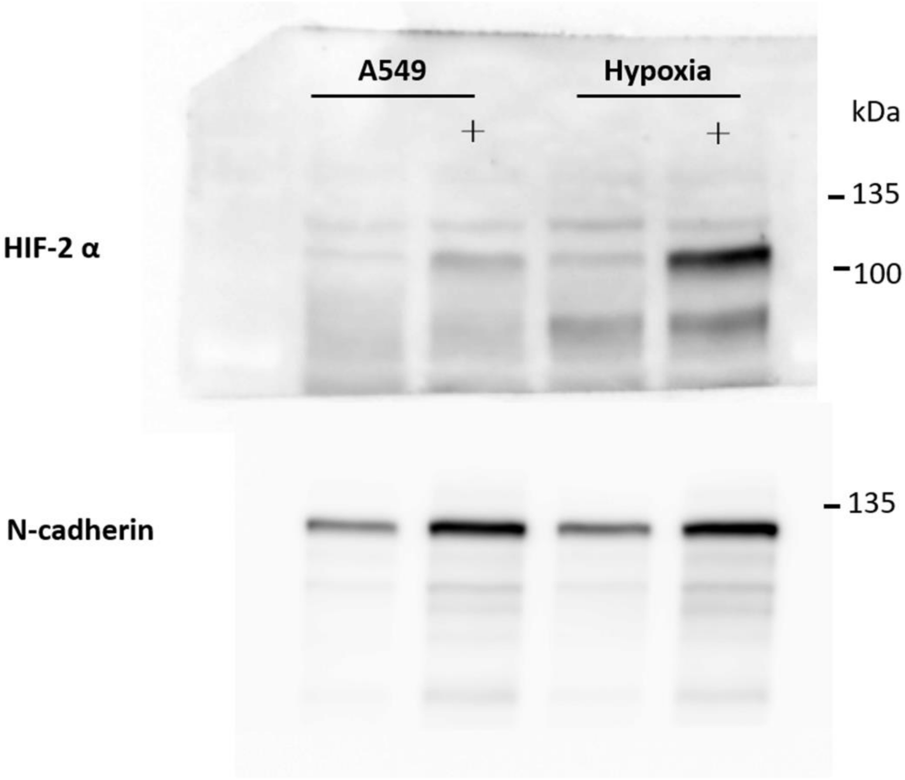

Supplement: Supplementary file 1 — Supplementary Information [file 41598_2017_8885_MOESM1_ESM.pdf]
